# Two-dimensional strain echocardiography for detection of cardiotoxicity in breast cancer patients undergoing chemotherapy

**DOI:** 10.15171/jcvtr.2017.04

**Published:** 2017-03-13

**Authors:** Mehrnoush Toufan, Leili Pourafkari, Leila Ghahremani Nasab, Ali Esfahani, Zohreh Sanaat, Alireza Nikanfar, Nader D Nader

**Affiliations:** ^1^Cardiovascular Research Center, Tabriz University of Medical Sciences, Tabriz, Iran; ^2^Department of Anesthesiology, University at Buffalo, Buffalo, NY, USA; ^3^Hematology and Oncology Research Center, Tabriz University of Medical Sciences, Tabriz, Iran

**Keywords:** Anthracycline, Breast Cancer, Strain Echocardiography

## Abstract

***Introduction:*** Two-dimensional (2D) strain echocardiography has emerged as a novel method for early diagnosis of myocardial dysfunction in patients receiving anthracycline chemotherapy. Certain myocardial segments might be more vulnerable for development of dysfunction.

***Methods:*** Sixty-three patients with breast cancer who were deemed amenable for anthracycline chemotherapy were prospectively studied from March 2013 to March 2015 in University Hospital settings. Global left ventricular (LV) ejection fraction (EF), fractional shortening and the strain over 17 segments of the LV were examined using 2-dimensional transthoracic echocardiography (TTE) before and after chemotherapy. More than 15% reduction in longitudinal peak systolic strain (LPSS) was considered significant.

***Results:*** The mean age of patients was 47 ± 10 years. LVEF was 59.7 ± 6.5% at baseline. Significant reduction of global LPSS was detected in 13% of patients. A significant LPSS reduction occurred in 32.4% of 1071 segments examined following chemotherapy. LPSS significantly decreased in 28% of apical segments, 31% of mid segments and 37% of basal segments. LPSS reduction occurred more frequently over the basal segments than all other segments (*P* = 0.031).

***Conclusion:*** Segmental pattern appears to exist in LPSS reduction following anthracycline therapy. As significant segmental decreases can be seen in the setting of unchanged global LPSS, segmental evaluation of LPSS might be a more accurate way for assessment of myocardial function.

## Introduction

The association between anthracyclines and cardiovascular complications has been established, and early diagnosis of these complications is pivotal in appropriate management of patients. Cardiotoxicity caused by chemotherapy constitutes a wide range from subclinical dysfunction to significant heart failure. Cardiotoxicity caused by anthracyclines is classified into acute, early onset chronic progressive and late onset chronic progressive.^1^ Acute cardiotoxicity occurs immediately after administration of anthracyclines in less than one percent of patients; in whom the transient reduction in myocardial contractility is often reversible. The risk of clinical cardiotoxicity increases with increasing dosage of anthracyclines.^2,3^ Studies suggest that the incidence of HF with 400 mg/m^2^, 550 mg/m^2^, and 700 mg/m^2^ dosages is 3%-5%, 7%-26%, and 18%-48%, respectively.^4^

Previous history of chemotherapy with anthracyclines; concomitant use of other cardiotoxic drugs (such as cyclophosphamide, trastuzumab, and paclitaxel), female sex, history of cardiovascular disease, old age and prolonged duration of treatment with anthracyclines are among the risk factors for development of anthracycline cardiotoxicity.^5^ Several mechanisms for anthracycline-induced cardiotoxicity have been proposed but formation of free radicals is the most important accepted mechanism.^6^ Left ventricular ejection fraction (LVEF) as assessed by conventional echocardiography is the routine method of evaluating left ventricular function. However, LVEF is a measure of global myocardial function and may lack the sensitivity to detect regional myocardial dysfunction. Additionally, it is limited by several technical factors.^7^ As earlier detection of cardiotoxicity is of paramount importance in these patients, two-dimensional (2D) strain echocardiography has emerged as a viable method for timely detection of subtle myocardial dysfunction. Remarkably strain values are influenced by blood pressure and heart rate.^8^ According to expert consensus, a relative percentage reduction of longitudinal peak systolic strain (LPSS) of >15% from baseline is considered of clinical significance in patients undergoing cancer treatment.^9^ Additionally, considering the segmental variations in normal values of regional strain, segmental specific normal range has been introduced.^8^ A few studies have focused on the regional changes in strain in these patients, however the results have been inconsistent.^7,10^

The objective of this research was to assess the global, regional and segmental changes of LPSS assessed through 2D strain echocardiography in adult patients with undergoing anthracycline chemotherapy for breast cancer.

## Patients and Methods

This is a prospective study conducted on female patients with biopsy-proven breast cancer undergoing chemotherapy with anthracyclines. Patients were recruited from the university-affiliated oncology hospital. A trained research team member obtained written informed consent from each participant. Female patients who were diagnosed with breast cancer and who were candidate for anthracycline therapy between March 2013 and March 2015 were considered for enrolment.

### Inclusion and exclusion criteria

Patients with a previous history of treatment with cardiotoxic drugs, non-sinus cardiac rhythm, history of myocardial infarction or symptomatic heart failure, previous cardiac surgery, and thoracic radiotherapy. Additionally, patients were excluded if they had significant valvular stenosis/regurgitation or LVEF less than 35% in the baseline echocardiography. Left ventricular (LV) systolic function was evaluated using both 2D-strain echocardiography and conventional echocardiography at baseline for each patient during the 72 hours prior to the initiation of chemotherapy. Patients were treated with doxorubicin 60 mg/m^2^ and cyclophosphamide 600 mg/m^2^. Patient characteristics including age, vital signs, height, weight, smoking status and comorbidities including hypertension and diabetes mellitus were recorded. After four cycle of chemotherapy, with three-week intervals between the consecutive cycles, (i.e. approximately 3 months after baseline study), participants were reassessed by conventional and longitudinal 2D-strain echocardiography and the study variables were measured and compared with baseline findings.

### Echocardiography

A single experienced cardiologist performed all echocardiography examinations by a single echocardiography machine (Vivid 7, GE, Norway). In all patients, enrolled in the study conventional echocardiography was performed on the left lateral decubitus position and cavity sizes were measured using the M-mode based on the parasternal long axis view. Left atrial volume index (LAVI) was measured in apical-2-chamber and apical-4-chamber views and was assessed using the area length method. The results were indexed based on body surface area of the enrolled patients. The mitral valve flow was measured by placing a sample volume Doppler at the tip of the mitral valve leaflets. Tricuspid annular plane systolic excursion (TAPSE) was measured by placing a curser of M mode such that one end was on the lateral side of the tricuspid valve and the other end was on right ventricular apex. The 2D strain assessment was carried out in a 2D environment, and AFI (Automated Functional Imaging) software was used in a multi-dimensional environment such that an apical-4-chamber view was captured in one cycle and the apical-4-chamber, apical-2-chamber, and apical-3-chamber views were also obtained simultaneously. The views were stored in the software and the 2D strain assessment was conducted using AFI offline.

Echocardiography variables included LV systolic function, LV end systolic volume (LVESV), LV end diastolic volume (LVEDV), LAVI, fractional shortening, E, A, E/A, (TAPSE), global, regional as well as segmental LPSS, global LPSS, segmental LPSS including apical, mid LPSS, and basal LPSS, as well as segmental LPSS for each of the 17 segments (6 basal, 6 mid and 5 apical) were evaluated prior and after treatment with anthracycline chemotherapy. A relative reduction of more than 15% in global LPSS between pre- and post-chemotherapy values was considered clinically significant according to the published expert consensus.^11^ Additionally, more than 10 percentage points decrease of LVEF to a value lower than the lower limit of normality was considered significant reduction.^9^

### Statistical analysis

The collected data were analyzed by SPSS version 22.0 (IBM^®^, Chicago, IL). Categorical variables were presented as frequencies and percentage, while continuous data were expressed as mean and standard deviation. Continuous variables were checked for normality of distribution by Kolmogorov-Smirnov. Fisher’s exact test was used for comparison of categorical variables. Continuous variables were compared by paired *t* test. Pearson’s correlation coefficients were used to explore correlation between LVEF and global and segmental LPSS. *P* value of ≤0.05 was considered statistically significant.

## Results

In this study 70 patients with biopsy-proven breast cancer fulfilled the inclusion criteria and were enrolled. One patient died during the study due to cancer-related complications, 4 patients withdrew consent and 2 patients were excluded according to the findings on their baseline echocardiography. Accordingly 63 patients completed the study. The mean age of patients included in the study was 47 ± 10 years. Seven patients had a previous medical history of diabetes, 14 patients had history of hypertension, and 4 patients had both of these comorbid conditions. There was no patient with a history of smoking. Thirty patients had right-sided tumors while 33 had left sided tumors. The mean systolic blood pressure of patients was 116±17 mm Hg, while their mean diastolic blood pressure was 72±13 mm Hg. The mean BMI of patients was 26.8 ± 2.8 kg/m^2^.

### Baseline echocardiographic findings of enrolled patients

Mean LVEF was 59.7 ± 6.5% at baseline. Mean fractional shortening was 32.6 ± 5.5%. Global LPSS was -19.1 ± 3.3 at baseline. Eight patients (13%) had grade I diastolic dysfunction (E/A < 0.75) and 4 patients (6%) had reduced RV systolic function (TAPSE < 16). In 39 patients (62%) LAVI was normal, and the rest had mild LA enlargement. Table 1 shows the baseline characteristics and echocardiographic findings of the study population.

**Table 1:** Baseline characteristics of 63 patients with breast cancer who underwent chemotherapy^a^

**Parameter**	**Frequency**	** **
Hypertension (%)	14	22%
Diabetes mellitus (%)	7	10.90%
Age (y)	63	46.6 ± 10.3
Systolic blood pressure (mm Hg)	63	116 ± 17
Diastolic blood pressure (mm Hg)	63	72± 13
Body mass index (kg/m^2^)	63	26.8 ± 2.8
Mitral inflow E velocity (cm/s)	63	71 ± 17
Mitral inflow A velocity (cm/s)	63	70 ± 12
E/A velocity ratio	63	1.03 ± 0.28
Deceleration time (ms)	63	190 ± 50
Tricuspid annular plane systolic excursion (mm)	63	21 ± 3
Maximum LA volume index (mL/m^2^)	63	32.6 ± 12.1
LV end-diastolic volume (mL)	63	87.5 ± 19.7
LV end-systolic volume (mL)	63	34.7 ± 11.4

^a^30 patients had left and 33 patients had right breast cancers.

**Figure 1:**
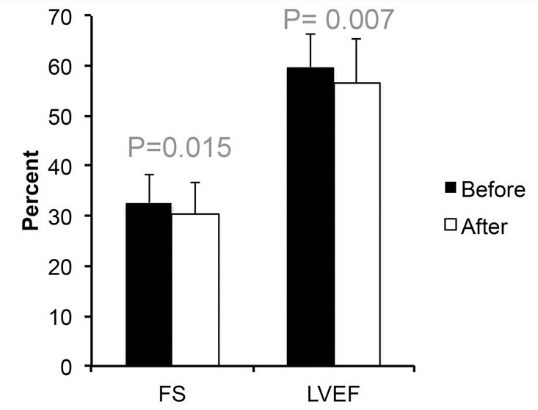
Left ventricular ejection fraction (LVEF) and fractional shortening (FS) prior and after chemotherapy.

After chemotherapy, mean LVEF decreased to 56.6 ± 8.2% (*P* = 0.006) and fractional shortening decreased to 30.4 ± 6.5% (*P* = 0.015). Figure 1 illustrates the comparison between the mean LVEF and fractional shortening prior and after chemotherapy. Additionally global LPSS decreased to -18.6±3.6 following treatment. In eleven patients (17.4%) a reduction of LVEF was observed following chemotherapy. The longitudinal 2D-strain echocardiographic findings before and after chemotherapy indicate that, significant reduction in LPSS (to less than -18%) detected in the basal anteroseptal (*P* = 0.013) and basal (*P* = 0.031) segments, but no significant difference was observed in other segments following chemotherapy when compared to pre-chemotherapy results. Pair-wise comparison of changes in strain of global and each segment of the left ventricle before and after chemotherapy is shown in Table 2. Additionally, the changes in LPSS were assessed in apical, mid and basal regions by averaging the values of contributing segments. Global as well as regional changes in LPSS according to the development of a significant reduction in LVEF were assessed. Table 3 shows the global as well as segmental changes in LPSS according to the development of a significant reduction in LVEF.

**Table 2:** Pair-wise comparison of changes in strain of global and various segments of the left ventricle before and after chemotherapy

	**Before chemotherapy**	**After chemotherapy**	**Mean ± STDEV changes**		**95% Confidence interval**		***t*** ** value**	***P*** ** value**
					**Lower**	**Upper**		
Global strain	-19.1 ± 3.3	-18.5 ± 3.6	-0.63	2.93	-1.37	0.10	-1.72	0.091
Apical cap	-19.7 ± 5.9	- 19.5 ± 6.1	-0.22	5.17	-1.52	1.08	-0.34	0.734
Apical septal	-20.8 ± 6.3	-20.5 ± 6.0	-0.22	5.51	-1.61	1.16	-0.32	0.750
Apical anterior	-19.1 ± 7.3	- 19.1 ± 6.8	0.03	8.08	-2.00	2.07	0.03	0.975
Apical lateral	-18.7 ± 6.6	-18.1 ± 6.2	-0.60	5.89	-2.09	0.88	-0.81	0.420
Apical inferior	-21.2 ± 5.6	-20.1 ± 6.9	-1.02	5.51	-2.40	0.37	-1.46	0.148
Mid anteroseptal	-18.6 ± 5.9	-17.3 ± 6.1	-1.33	5.72	-2.77	0.11	-1.85	0.069
Mid anterior	-17.8 ± 7.9	-15.9 ± 10.7	-1.86	10.13	-4.41	0.70	-1.45	0.151
Mid anterolateral	-17.2 ± 7.4	-18.2 ± 6.3	0.92	6.51	-0.72	2.56	1.12	0.266
Mid inferolateral	-17.1 ± 5.8	-16.4 ± 7.3	-0.67	7.97	-2.67	1.34	-0.66	0.509
Mid Inferior	-18.0 ± 6.1	-16.8 ± 7.4	-1.16	8.72	-3.36	1.04	-1.05	0.296
Mid inferoseptal	-19.7± 6.3	-19.2 ± 6.1	-0.51	6.10	-2.04	1.03	-0.66	0.511
Basal anteroseptal	-16.2 ± 5.4	-13.5 ± 9.0	-2.70	8.42	-4.82	-0.58	-2.54	0.013
Basal anterior	-16.1 ± 8.9	-14.5 ± 10.4	-1.62	9.99	-4.13	0.90	-1.29	0.203
Basal anterolateral	-18.3 ± 8.5	-17.5 ± 10.1	-0.78	9.55	-3.18	1.63	-0.65	0.520
Basal inferior	-18.2 ± 9.4	-16.3 ± 10.7	-2.14	11.85	-5.13	0.84	-1.44	0.156
Basal inferolateral	-17.3 + 8.5	-15.1 ± 10.5	-1.98	11.55	-4.89	0.92	-1.36	0.178
Basal inferoseptal	-17.9 ± 6.8	-16.6 ± 9.6	-1.37	8.47	-3.50	0.77	-1.28	0.206

**Table 3:** Association between significant drop (greater than 10%) in left ventricular ejection fraction (LVEF) and the presence of significant decrease in longitudinal peak systolic strain (> 15%) measured globally and various regions of the left ventricle

**Parameter**	**No change in LVEF (n=52)**	**Drop in LVEF>10% (n=11) **	**Odds ratio (95% CI)**	***P*** ** value**
Global stress ≥15%	5 (9.6%)	3 (27.3%)	3.53 (0.70–17.74)	0.137
Regional stress ≥ 15%				
Apical region	12 (23.1%)	6 (54.5%)	4.00 (1.04–15.44)	0.062
Mid-papillary region	7 (13.5%)	3 (27.3%)	2.41 (0.51–11.33)	0.360
Basal region	13 (25.0%)	4 (36.4%)	1.71 (0.43–6.81)	0.469

We did not observe a significant correlation between the results of LPSS and presence of diabetes and hypertension among the participants. Interestingly in 8 patients out of 11 patients with significant LVEF reduction did not have a significant decrease in global LPSS. However, significant decrease in LPSS was observed in several segments of these patients.

**Figure 2:**
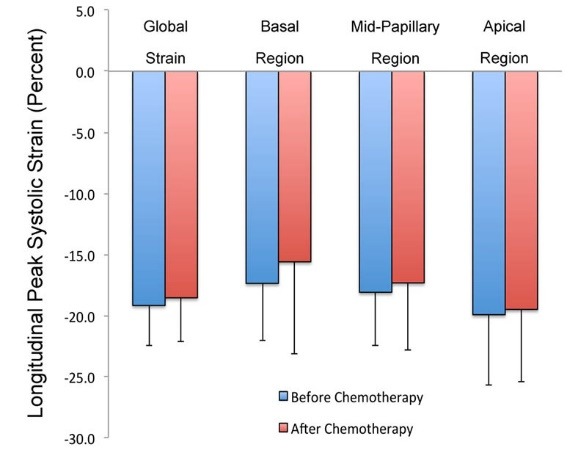
Two-dimensional strain echocardiography values at before and after chemotherapy.

Concerning the significant reduction in LPSS (relative change of reduction >15%), based on the longitudinal 2D strain echocardiography results, eight patients (13%) showed a significant reduction in global LPSS. Moreover, the assessment of longitudinal 2D-strain of apical segments, in 18 cases (28.6%), mid segments, in 10 cases (16%) and basal segments, in 17 cases (27%) demonstrated significant reduction (>15% relative decrease). Significant reductions in the 89 out of 315 apical segment (28%), 117 out of 378 mid segments (31%) and 141 out of 378 basal segment (37.3%) were noted. Assessing all 17 segments in 63 recruited patients (i.e. 1071 segments altogether) indicated a significant reduction in 347 (32%) segments. Figure 2 shows mean global and segmental LPSS values before and after anthracycline therapy.

One patient (1.6%) required hospitalization for treatment of acute heart failure. This particular patient had a LVEF of 35% and reduction in her global LPSS from -23% to -11%.

Additionally, the correlation of percentage changes in regional and global LPSS and changes in LVEF the respective baseline values were evaluated and is shown in Table 4. As shown for LVEF, global LPSS has the highest albeit fair correlation (r = 0.441). Global LPSS has the highest correlation with the LPSS measures at mid papillary segments (r = 0.647).

**Table 4:** Correlation of changes in regional and global strain to changes in left ventricular ejection fraction expressed in percent changes from their respective baseline values

		dEF	dGLs	dAPR	dMPR	dBR
dEF	Correlation Coefficient (R-Value)	1.00	0.441^**^	0.216	0.365^**^	0.272
	*P* value (2-tailed)		0.0003	0.0888	0.0033	0.0309
dGLs	Correlation Coefficient (R-Value)	0.441^**^	1.00	0.540^**^	0.647^**^	0.616^**^
	*P* value (2-tailed)	0.0003		<0.0001	<0.0001	<0.0001
dAPR	Correlation Coefficient (R-Value)	0.216	0.540^**^	1.00	0.558^**^	0.343^**^
	*P* value (2-tailed)	0.0888	<0.0001		<0.0001	0.0060
dMPR	Correlation Coefficient (R-Value)	0.365^*^	0.647^**^	0.558^**^	1.00	0.815^**^
	*P* value (2-tailed)	0.0033	<0.0001	<0.0001		<0.0001
dBR	Correlation Coefficient (R-Value)	0.272	0.616^**^	0.343^**^	0.815^**^	1.00
	*P* value (2-tailed)	0.0309	<0.0001	0.0060	<0.0001	

*Correlation is significant at the 0.01 level (2-tailed). **Correlation is significant at the 0.001 level (2-tailed). Abbreviations: dEF: Percent deterioration in left ventricular ejection fraction; dGLs: Percent deterioration of left ventricular global strain; dAPR: Percent deterioration of left ventricular strain in apical region; dMPR: Percent deterioration of left ventricular strain in midpapillary region; dBR: Percent deterioration of left ventricular strain in basal region.

## Discussion

Anthracyclines and relevant compounds are among the most common causes of drug-induced cardiotoxicity and timely recognition of myocardial damage in patients receiving these agents is essential. Yeh et al introduced echocardiography as a useful non-aggressive alternative method for the assessment of heart function and diagnosis of cardiotoxicity caused by administration of anthracyclines.^12^ Additionally, Doppler echocardiography was suggested to be a useful tool for determination of the hemodynamic status, especially when increased pulmonary arterial pressure is suspected. In the study published by Tassan-Mangina et al in 2006, 20 patients receiving doxorubicin were examined by echocardiography before, 1-3 months and 3-5 years after chemotherapy. In this study late reduction LVEF despite an initially normal values was commonly reported after anthracycline therapy.^13^ Subtle changes in myocardial performance may not be readily identifiable by LVEF. Moreover, the difficulty of LV afterload assessment by echocardiography adversely affects specificity of LVEF. Considering these limitations, 2D strain echocardiography has gained popularity for early detection of cardiotoxicity associated with chemotherapy. Jurcut et al introduced the application of strain rate imaging for timely detection of subtle myocardial injury in a study performed on 16 patients undergoing anthracycline therapy.^14^ It has been suggested that the changes in strain could be regional and segmental, though this has been a matter of controversy.^15^ According to the recommendations, modified Simpson method is the preferred method for measuring LVEF on two-dimensional echocardiography. In this recommendation calculation of LVEF is to be combined with the wall motion score index.^16^

Wall motion score index measurement based on the 17-segment model is considered to be a more sensitive method when compared to LVEF for evaluation of the extent of injury induced by anthracyclines. Likewise, myocardial deformation can be measured through Doppler tissue imaging or 2D Speckle tracking echocardiography.^16^ Regional wall motion abnormalities particularly in the apical segments in patients receiving anthracycline treatment have been recognized for a long time.^17^ In one study on pediatric population, regional changes in basal, and mid sections and basal anterolateral, mid anterior, mid inferior, segments were reported.^10^ Additionally, segmental reductions in LPSS in mid and apical segments were found to have a reasonable predictive value for reduction of LVEF in follow-up.^10^ In another study on 52 women with breast cancer significant reductions of LPSS was noted in all but apical lateral segment in the echocardiography exam performed 1 week after termination of anthracycline chemotherapy.^7^ In accordance with our findings, these authors have also pointed out the possibility of regional heterogeneity in the development of myocardial dysfunction, though the technical limitation pertinent to image quality could have also contributed to this observation.^7^

In accordance with previous studies, we also report that longitudinal 2D-strain echocardiography, is a valuable tool in diagnosis of anthracycline-induced toxicity.^7,10^ However, we identified patients in whom despite a decrease in LVEF, global longitudinal peak systolic strain failed to show any significant changes. We observed that these patients had higher heart rates in the second exam. We also noticed that these patients had significant strain reduction in several segments yet their effect might have been offset by the changes in LPSS of other segments in the opposite direction. We assume that global LPSS may not be influenced if the reduction of LPSS in some segments is accompanied by an increase in the value in other segments. For further evaluation, we did a correlation analysis and we observed that changes of LPSS in mid papillary region has the highest correlation with the global LPSS. The contribution of each segment to the global LPSS may not be equal and as such involvement of various segments/regions could translate to dissimilar effects on global value of LPSS. As such, in these cases global LPSS of these patients could be normal, which highlights the need for not only global but also a segmental assessment of the values. This is consistent with the histologic reports of scattered focal myocardial fibrosis in these patients.^18^ We acknowledge that this observation could be due to the presence of tachycardia following chemotherapy. Higher heart rates can adversely affect the clarity of endocardial borders in certain views and therefore, measurement errors are more likely to happen.

In the present study, the conventional and longitudinal 2D-strain echocardiography methods were used to examine the toxic effects of anthracyclines. The results of longitudinal 2D-strain echocardiography were not significantly influenced by the presence of diabetes or hypertension. Significant LPSS reduction was detected in the basal anteroseptal and all-basal segments, but no significant difference was observed in other segments. Additionally, segmental changes were observed in patients with no reduction of global LVEF, which denote the presence of a regional pattern in the occurrence of myopathy.

We conclude that the longitudinal 2D-strain echocardiography findings obtained before and after chemotherapy indicate a segmental pattern in myocardial dysfunction. 2D-strain echocardiography is a useful tool for timely diagnosis of myocardial dysfunction, though global assessment may be subject to limitations. Considering the observed regional pattern in myocardial involvement, segmental evaluation of longitudinal peak systolic strain might be a more accurate way of detecting early myocardial dysfunction. Further studies enrolling larger number of patients supplemented with histo-pathological evidences are much anticipated.

## Limitations

The study is limited by the relatively small sample size and lack of long-term follow-up. Additionally, the definition of significant decrease of >15% has been proposed for global LPSS. Accordingly, since correspondent values for each segment has not been validated the assumption might not necessarily hold true for segmental or regional values. Furthermore, a single echocardiographist performed all the measurements and we have not evaluated the intra-observer variability.

## Competing interests

The authors declare that they have no competing interests.

## Ethical Approval

Institutional review board and ethics committee approved the study protocol.

## Acknowledgments

We would like to express our sincere thanks to Dr. Soheyla Nazarnia, and Ms. Maryam Ghahremani Nasab for their invaluable helps with data collection and editing the paper.
